# EZH2, JMJD3, and UTX epigenetically regulate hepatic plasticity inducing retro-differentiation and proliferation of liver cells

**DOI:** 10.1038/s41419-019-1755-2

**Published:** 2019-07-08

**Authors:** Natalia Pediconi, Debora Salerno, Leonardo Lupacchini, Annapaola Angrisani, Giovanna Peruzzi, Enrico De Smaele, Massimo Levrero, Laura Belloni

**Affiliations:** 1 Center for Life Nano Science@Sapienza, Istituto Italiano di Tecnologia, Rome, Italy; 2 IRCCS San Raffaele Pisana, Rome, Italy; 3 Dept. Molecular Medicine, Sapienza University of Rome, Rome, Italy; 4 Dept. of Experimental Medicine, Sapienza University of Rome, Rome, Italy; 5 Cancer Research Center of Lyon (CRCL), UMR INSERM U1052 - CNRS, 5286 Lyon, France; 6 Dept. of Internal Medicine and Medical Specialties, Sapienza University of Rome, Rome, Italy; 7 Hepato-Gastroenterologie, Hopital de la Croix-Rousse, Hospices Civils de Lyon, Lyon, 69004 France

**Keywords:** Methylation, DNA methylation

## Abstract

Modification of histones by lysine methylation plays a role in many biological processes, and it is dynamically regulated by several histone methyltransferases and demethylases. The polycomb repressive complex contains the H3K27 methyltransferase EZH2 and controls dimethylation and trimethylation of H3K27 (H3K27me2/3), which trigger gene suppression. JMJD3 and UTX have been identified as H3K27 demethylases that catalyze the demethylation of H3K27me2/3, which in turns lead to gene transcriptional activation. EZH2, JMJD3 and UTX have been extensively studied for their involvement in development, immune system, neurodegenerative disease, and cancer. However, their role in molecular mechanisms underlying the differentiation process of hepatic cells is yet to be elucidated. Here, we show that EZH2 methyltransferase and JMJD3/UTX demethylases were deregulated during hepatic differentiation of human HepaRG cells resulting in a strong reduction of H3K27 methylation levels. Inhibition of JMJD3 and UTX H3K27 demethylase activity by GSK-J4 epi-drug reverted phenotype of HepaRG DMSO-differentiated cells and human primary hepatocytes, drastically decreasing expression of hepatic markers and inducing cell proliferation. In parallel, inhibition of EZH2 H3K27me3 activity by GSK-126 epi-drug induced upregulation of hepatic markers and downregulated the expression of cell cycle inhibitor genes. To conclude, we demonstrated that modulation of H3K27 methylation by inhibiting methyl-transferase and dimethyl-transferase activity influences the differentiation status of hepatic cells, identifying a possible new role of EZH2, JMJD3 and UTX epi-drugs to modulate hepatic cell plasticity.

## Introduction

Chromatin remodeling represents a highly dynamic and reversible process in which there is continual laying down and removal of modifications of histones N-terminal tails by chromatin-remodeling enzymes. In particular, the *N*-terminal tails of histones contain lysine (K) and arginine (R) residues that can undergo different posttranslational modifications. Try- or di-methylation of lysine 27 (H3K27me2/3) and lysine 9 on histone H3 (H3K9me3) are hallmarks of silenced chromatin whereas methylation of lysine 4 on histone 3 (H3K4me3) is a marker of active transcription^1^. Classification of histological samples based on H3K27 acetylation and H3K27me3 identified an aggressive subgroup of hepatocellular carcinoma (HCC), and could serve as a prognostic marker for HCC^2^.

Enhancer of Zeste Homolog 2 (EZH2) methyltransferase is a component of Polycomb Repressive Complex 2 (PRC2) complex and functions as a histone methyltransferase that specifically induces H3K27me3 to the targeted genes. PRC2 has been shown to deregulate gene expression promoting cancer cell growth and proliferation and inhibiting differentiation process^3,4^. Indeed, recent work suggested that modulation of EZH2 activity is critical in regenerative medicine^5^. Furthermore, it has been shown that EZH2 is essential for expansion of hepatic progenitor population and its loss of function results in decreased expression of hepatic differentiation marker genes^6,7^.

Since H3K27me3 methylation is associated with gene repression, removal of these marks by histone demethylases such as Ubiquitously transcribed Tetratricopeptide repeat on chromosome X (UTX) and Jumonji Domain Containing protein 3 (JMJD3) lead to transcriptional activation^8^. UTX and JMJD3 are closely related histone demethylases, encoded by KDM6A and KDM6B genes respectively, and act specifically on H3K27me2/3^9^. Deletion of KDM6A causes embryonic lethality^10^. It has been demonstrated that UTX has an essential role during development of different tissue^11,12^. Although the decrease of UTX expression promotes proliferation in many cellular contexts, the role of UTX in cancer seems to be rather tissue and cell specific^13^. In agreement with this observation, overexpression of UTX in breast cancer promotes proliferation and invasion^14^.

JMJD3 demethylase enzyme regulates transcriptional activation of genes involved in several biological processes^15^. It has been hypothesized a role of JMJD3 in removal of H3K27me3 mark from promoters involved in reprogramming of adult bone marrow progenitor cells to hepatocytes^16^. It has been demonstrated that decreased expression of JMJD3 which reduces H3K27 demethylation at the INK4A–ARF tumor suppressor locus^8^ might contribute to the development of some human cancers, including lung and liver carcinomas, as well as diverse hematopoietic malignancies. Moreover, a recent work has demonstrated that JMJD3 is highly expressed in primary HCC cells and its overexpression induced EMT and invasive migration in HCC cells^17^. However, the role of the demethylases UTX/JMJD3 in liver cancer cells remains to be further elucidated.

KDM6B and KDM6A play an important role in endoderm differentiation from human ESCs and knockdown of KDM6A or KDM6B impairs endoderm differentiation^18^. Meanwhile transient expression of the catalytic domain of JMJD3 significantly accelerates human pluripotent stem cells differentiation into hepatic or muscle cells^19^.

To better understand the role of EZH2, JMJD3 and UTX in hepatic differentiation and proliferation, we took advantages of the HepaRG cell model^20^. In this study we treated differentiated HepaRG and PHH with GSK-126^21^ and GSK-J4^22^, two small inhibitors of H3K27me3 methylase (EZH2) and demethylases (UTX/JMJD3) respectively, able to regulate H3K27me3 levels. We investigated gene expression profiles of RNAseq based on dHepaRG treated or not with GSK-J4 demonstrating that modulation of H3K27me3 levels influences hepatic plasticity inducing retro-differentiation and proliferation.

## Materials and methods

### Cell culture and treatments

Human hepatic HepaRG cells were seeded at low density in proliferation medium (William’s E medium with GlutaMAX (Gibco), supplemented with 10% FBS (Hyclone II GE), 1% penicillin/streptomycin (Sigma), 5 µg/mL insulin (Sigma), 0.5 µM hydrocortisone hemisuccinate (Sigma)). After 1 week of culture, at 100% confluence, cells were shifted into the differentiation medium (William’s E medium with GlutaMAX (Gibco), supplemented with 10% FBS (Hyclone II GE), 1% penicillin/streptomycin (Sigma), 5 µg/mL insulin (Sigma), 50 µM hydrocortisone hemisuccinate and 2% DMSO (Sigma)) for 2 more weeks to obtain confluent differentiated cultures. Human HepaRG cells show hepatic progenitor features and are able to differentiate into both hepatocyte and biliary lineages. Human Primary Hepatocytes were purchased from Life Technologies (n. catalog. HMCPIS) and were cultured as manufacturer’s protocol. Hepatocellular carcinoma HepG2 cells were cultured in DMEM supplemented with 10% fetal bovine serum (FBS) and 1% penicillin/streptomycin (Sigma). Cells were treated with GSK-J4 (25 µM) and/or with GSK-126 (10 µM) (Selleckchem catalog. No. S7070 and S7061 respectively), for the indicated time; GSK-J4 and GSK-126 were diluted in proliferation medium for pHepaRG treatments and in differentiation medium for dHepaRG treatments. Compounds cytoxicity was tested by Fixable Viability Dye eFluor 780 (affymetrix eBioscience 65-0865) used to irreversibly label dead cells (Supplementary Methods and Figs. S1 and S2).

### ELISA assay

The expression levels of Albumin secreted from GSK-J4 treated dHepaRG and PHH cells were detected by enzyme-linked immunosorbent assay, Albumin ELISA kit from Abcam (Ab108788). Cell culture media was centrifuged at 1000 × *g* for 10 min to remove debris and supernatants were collected to perform standard Elisa as manufacturer’s protocol.

### Proliferation assay

Proliferating pHepaRG cells treated or not with GSK-126 and GKS-J4 for 72 h were fluorescent labeled (5 h) with the Click-iT® EdU Alexa Fluor® 488 HCS Assay (Thermofisher) as manufacturer’s instruction.

### CYP activity assay

CYP3A4 enzymatic activity was measured by the P450-Glo Assay (Promega) luminescent method as manufacturer’s protocol.

### Immunofluorescence

Cells were fixed with 4% paraformaldehyde followed by permeabilization with 0.2% Triton X-100. Cells were incubated with anti-Ki-67 for 1 hour or CK19 antibody overnight (Table S4). Nuclei were counterstained with Hoechst and observed under a fluorescence microscope. The cell count was performed by ImageJ software.

### Scratch wound migration assays

A scratch wound (1–1.5 mm in width) was made by scraping the cell monolayer of proliferating or differentiated HepaRG cells with a sterile tip. After washing twice (PBS 1×), wounded cultures were treated with GSK-J4 (25 µM) and/or with GSK-126 (10 µM). At T0, 24, 48 and 72 h after scratching, cells were photographed under an inverted phase-contrast microscope and the migratory area covered was assessed using the ImageJ software.

### Immunoblotting

Cells were lysed in NET buffer (50 mM Tris–HCl pH 7.5, 150 mM NaCl, 0.1% NP-40, 1 mM EDTA pH 8) and immunoblotted with the antibodies listed on Table S4.

For histone acid extraction we performed cell lysis with a specific kit from Abcam (ab113476). Proteins of interest were detected with HRP-conjugated anti-mouse/rabbit/goat IgG antibodies from Santa Cruz Biotechnology and visualized with the Pierce ECL Western blotting substrate (ThermoScientific), according to the provided protocol. Densitometric analysis was performed by ImageJ software.

### Chromatin Immunoprecipitation (ChIP)

Chromatin from dHepaRG cells was immuno-precipitated with antibodies listed on Table S4. Chromatin immunoprecipitated was analyzed by qPCR using fluorescent dye SYBR Green in a Light Cycler 480 instrument (Roche Diagnostics). List of primers are listed in Supplementary Table S3.

### FACS analysis

See Supplementary Methods.

### RNA extraction and sequencing analysis

Total RNAs from HepaRG cells were isolated using TRIzol reagent (Invitrogen). cDNA was synthesized using a Maxima-H-minus-First-Strand-cDNA Synthesis Kit (Thermoscientific) and analysed with gene specific primers by qPCR using the fluorescent dye SYBR Green in a Light Cycler 480 instrument (Roche Diagnostics). GAPDH was used as internal control for normalizing equal loading of the samples. Complete list of primers in Supplementary Table S3.

RNA sequencing was performed by IGATECH (Udine, Italy)^23–27^. The datasets generated by RNAseq and analysed during the current study are available at NCBI website with n.project BioProject PRJNA508878). Library preparation, sequencing and bioinformatics analysis are described in Supplementary Methods.

### EDU assay

Click-iT™ EdU Alexa Fluor™ 488 Imaging Kit (Life Technologies, C10337) is optimized to label proliferating cells and the assay was performed 2 h after EDU incorporation in accordance with manufacturer’s instructions.

### Statistics

P-values w*ere* determined using the 2-tailed Student’s *T*-test: *0.01 ≤ *P* < 0.05; **0.001 ≤ *P* < 0.01; ***P < 0.001. Results are expressed as mean of three independent experiments, bars indicate Standard Deviation. The cell cycle analysis was calculated applying the Dean/Jett/Fox algorithm of the FlowJo software.

## Results

### EZH2, JMJD3, and UTX are modulated during hepatic differentiation

**Fig. 1:**
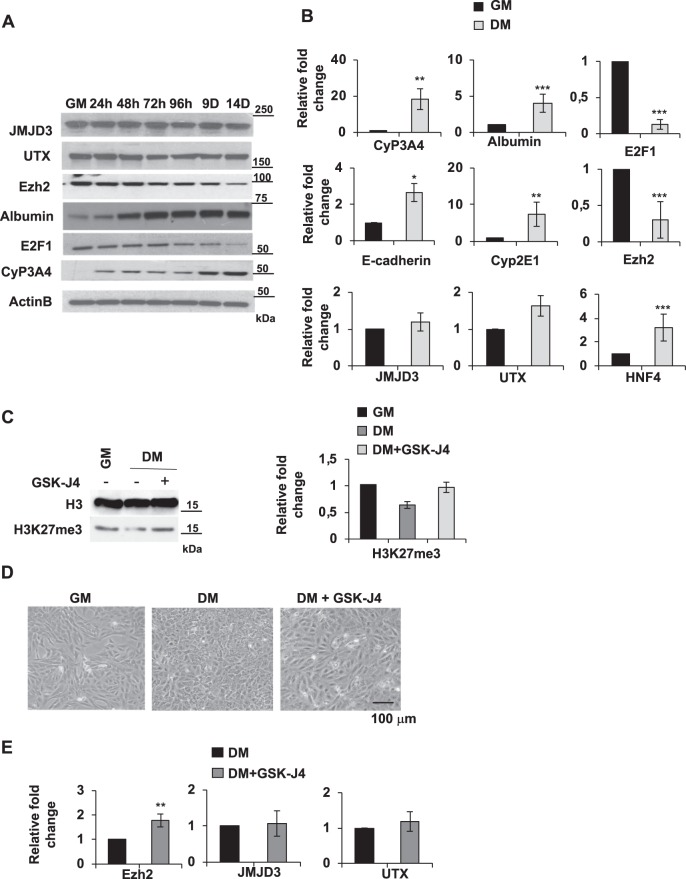
EZH2, JMJD3 and UTX are deregulated during hepatic differentiation. **a** Total protein lysates were extracted from proliferating HepaRG (GM) and differentiated HepaRG cells for the indicated time. Cells were harvested and the immunoblotting analysis was performed using specific antibodies (Table S4). **b** Total RNAs were extracted from pHepaRG (GM) and dHepaRG (DM) cells, qPCR analysis was performed using specific primers (Table S3). Amplification of GAPDH transcripts was used to normalize equal loading of each RNA samples. Histograms show the fold induction of DM versus GM. **c** Nuclear acid protein lysates from pHepaRG (GM) and dHepaRG cells (DM) treated or not with GSK-J4 for 48 h were analyzed by Immunoblot (left panel) with the indicated antibodies (Table S4); right panel: densitometric analysis is expressed as fold induction (FI) of DM, DM + GSK-J4 versus GM cells. **d** Optical microscope images of HepaRG cells treated as in (**a**). **e** Total RNAs were extracted from dHepaRG (DM) cells treated or not with GSK-J4 for 48 h and qPCR analysis was performed using specific primers (Table S3). Amplification of GAPDH transcripts was used to normalize equal loading of each RNA samples. Histograms show the fold induction of treated cells (GSK-J4) versus untreated (DM). All results are expressed as fold induction (mean) from three independent experiments, bars indicate S.D.; Asterisks indicate *P*-value: *0.01 ≤ *P* < 0.05; **0.001 ≤ *P* < 0.01; ***P < 0.001

In order to investigate the role of histone methylation, during the process of hepatic differentiation, we first evaluated protein and transcript levels of methyltransferase EZH2 and demethylases JMJD3 and UTX in differentiating HepaRG cells. Interestingly, we observed that EZH2 transcripts and protein levels were decreased during the hepatic differentiation (Fig. 1a, b). Conversely, demethylases JMJD3 and UTX did not show any significative difference both at the transcript and at the protein levels between differentiated (DM 14 days) and proliferating HepaRG cells (GM) (Fig. 1a, b). The transcription factor E2F1, which has been described to bind and activate EZH2 promoter^28,29^, is strongly decreased during differentiation paralleling EZH2 levels (Fig. 1a, b), suggesting a possible role of E2F1 in the transcriptional regulation of EZH2 during hepatic differentiation. As expected, we could show that the liver-specific proteins Cyp3A4 and Albumin already increased at the early stage of the differentiation process (Fig. 1a). Moreover, in cells differentiated for 14 days (DM) transcript levels of hepatic genes Cyp3A4, Albumin, Cyp2E1, E-cadherin and HNF4 were upregulated as compared to proliferating cells (GM) (Fig. 1b). As shown in Fig. 1c, we observed that H3K27me3 protein levels are reduced after 14 days differentiated HepaRG cells (DM-, third lane) as compared to proliferating cells (GM−, first lane). Importantly, inhibition of JMJD3 and UTX activity with the cell permeable drug GSK-J4 after 14 days dHepaRG cells led to a restoration of H3K27me3 levels (DM+, third lane), reaching levels comparable to proliferating cells (GM−, first lane) (Fig. 1c).

By optical microscope analysis we observed that treatment with GSK-J4 (DM + GSK-J4) was able to induce morphology changes of dHepaRG cells from a differentiated phenotype (DM) into a phenotype similar to proliferating cells (Fig. 1d). Moreover, we observed that treatment with GSK-J4 didn’t affect JMJD3 and UTX transcripts levels in dHepaRG cells (Fig. 1e), demonstrating that GSK-J4 is able to regulate their activity, but not their expression. Conversely, EZH2 transcript levels were slightly, but significantly upregulated (Fig. 1e) suggesting a feedback regulation between methylase and demethylase enzymes. These data show that H3K27me3 levels decreased in dHepaRG cells and suggest a central role of JMJD3 and UTX demethylases activity in the hepatic differentiation.

### Gene expression profiling of dHepaRG cells treated with GSK-J4

**Fig. 2:**
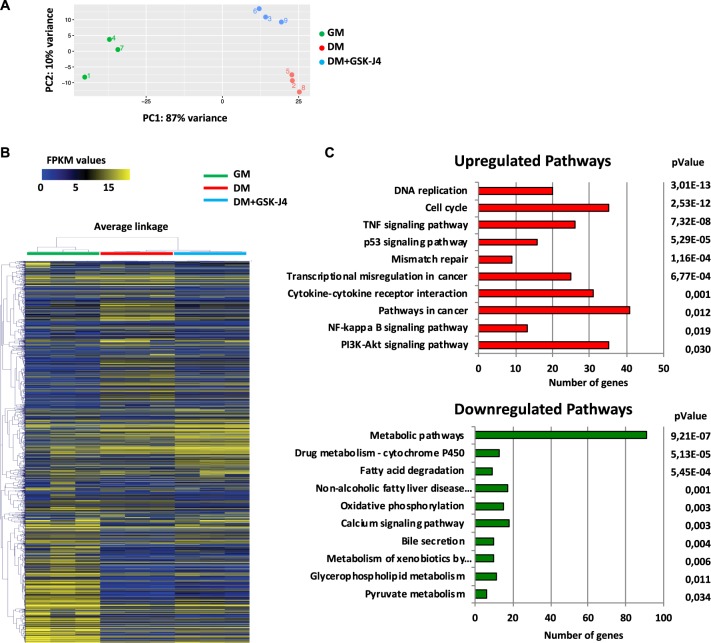
RNA sequencing analysis of GSK-J4 treated HepaRG cells. **a** Principal Component Analysis (PCA) of total RNA extracted from pHepaRG (GM) and dHepaRG cells (DM) treated or not for 24 h with GSK-J4 (DM + GSK-J4). **b** Heat-map analysis showing gene expression levels in HepaRG cells treated as in (**a**). FPKM (Fragments Per Kilobase Million) values are indicated with blue and yellow colors. **c** KEGG analysis of biological pathways of the up and down-regulated genes in GSK-J4 treated versus untreated dHepaRG cells. RNA-seq was performed on three independent experiments

To further study the role of JMJD3 and UTX in hepatic differentiation we performed gene expression profiling by total RNA sequencing analysis in pHepaRG cells and dHepaRG cells treated or not with GSK-J4. Principal Component Analysis (PCA) showed that differentiated cells were clustered together, completely separated from the proliferating cells, as expected (Fig. 2a). Interestingly, the expression profiles of dHepaRG cells treated with GSK-J4 deviated from those of differentiated control cells and were closer to those of proliferating cells. Same evidences are shown by hierarchical clustering in Heat Map analysis (Fig. 2b). To determine the signaling pathways associated with the differential expressed gene signature, we performed Gene Ontology (GO) by KEGG analysis. Interestingly, we observed that GSK-J4 was able to stimulate DNA replication, cell cycle and PI3K-Akt signaling together with survival pathways such as p53 signaling and Mismatch repair (Supplementary excel file). Besides these pathways involved in growth and proliferation we found activation of several inflammatory genes involved in both pathways such as TNF signaling and NF-kappa B signaling pathways (Fig. 2c upper panel and Table S1).

Together with the upregulated pathways, the analysis of GSK-J4 profiles versus control cells revealed several downregulated pathways, such as metabolic pathway, NAFLD, Fatty acid degradation and drug metabolism-cytochrome P450 (Fig. 2c lower panel). Many genes from GO analysis involved in these metabolic pathways are also related to hepatic differentiation (Supplementary excel file)^20,30^. These results indicate that GSK-J4 inhibition of JMJ3/UTX influences hepatic plasticity re-inducing proliferation of dHepaRG cells and decreasing expression of liver marker genes.

### GSK-J4 inhibition of JMJD3 and UTX H3K27me3 demethylase activity led to retro-differentiation of dHepaRG and PHH cells

**Fig. 3:**
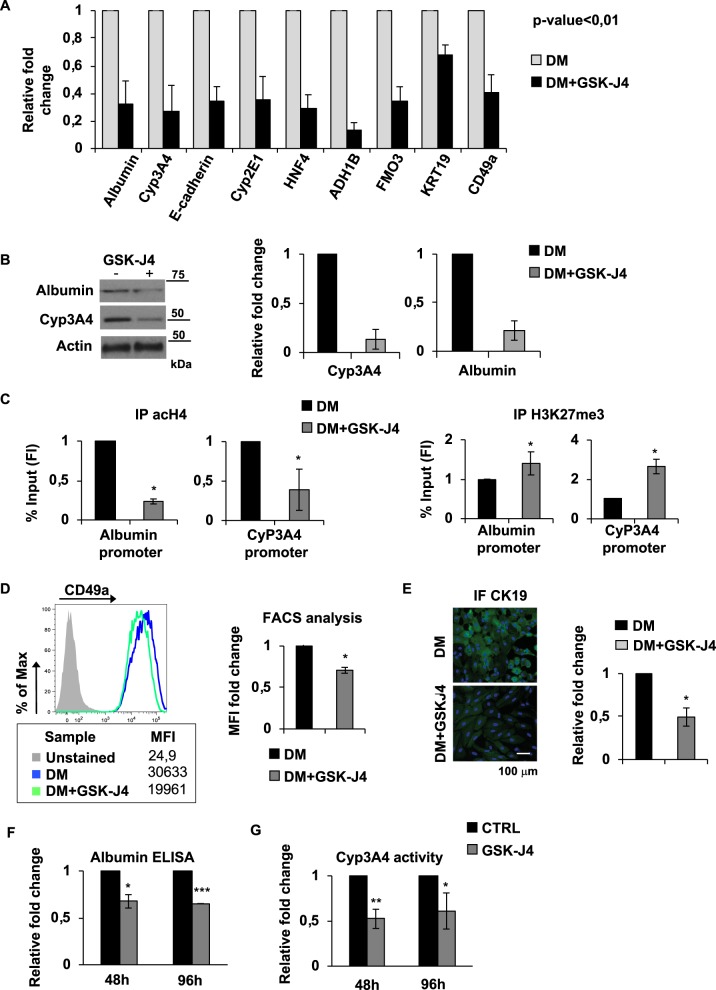
GSK-J4 treatment induced retro-differentiation of dHepaRG and PHH cells. **a** Total RNAs were extracted from dHepaRG cells untreated (DM) or treated with GSK-J4 for 24 h and qPCR analysis was performed using indicated primers (Table S3). Amplification of GAPDH transcripts was used to normalize equal loading of each RNA samples. Histograms show the fold induction of treated cells (GSK-J4) versus untreated (DM). **b** Total protein lysates were extracted from dHepaRG cells and were harvested 48 h after GSK-J4 treatment and immunoblotted with the indicated antibodies (Table S4). Analysis of Cyp3A4 and Albumin were showed and actin was used as control, histograms show densitometric analysis expressed as fold induction (FI) of DM + GSK-J4 versus DM (right panel). **c** Cross-linked chromatin was extracted from dHepaRG cells treated for 48 h with GSK-J4 and immunoprecipitated with a relevant control IgG or specific anti-AcH4 and anti-K27me3 antibodies (respectively left and right panels). Immunoprecipitated chromatin samples were analyzed by qPCR using Albumin and Cyp3A4 promoter selective primers. Percentage of input was calculated by Delta Ct analysis and it expressed as fold induction of DM versus GM. **d** FACS analysis of dHepaRG cells treated or not with GSK-J4 for 48 h and stained with anti CD49a. FACS plot is a representative example (left panel) and table shows MFI (mean fluorescence intensity), (lower panel). Histograms represent MFI expressed as fold induction of DM + GSL-J4 versus DM (right panel). **e** Immunofluorescence staining with CK19 antibody and Hoechst of dHepaRG cells treated or not with GSK-J4 for 48 h (left panel). Histograms represent relative number of CK19 positive cells (green) over total number of cells (blue) (right panel). **f** Supernatants from PHH treated for 48 or 96 h with GSK-J4 were analyzed by ELISA to quantify levels of secreted Albumin. Histograms show fold induction of treated (GSK-J4) versus control cells (CTRL). **g** CyP3A4 enzymatic activity from cells treated as in (**f**) were quantified by P450-GLO assay. Histograms show fold induction of treated (GSK-J4) versus control cells (CTRL). All results are expressed as fold induction from three independent experiments, bars indicate S.D.; Asterisks indicate *P*-value: *0.01 ≤ *P* < 0.05; **0.001 ≤ *P* < 0.01; ***P < 0.001

To validate RNAseq. results, we analysed by qPCR the expression of selected genes from KEGG-GO analysis downregulated after GSK-J4 treatment (Fig. 2c lower panel). We confirmed that inhibition of JMJD3 and UTX by GSK-J4 in dHepaRG cells was able to strongly reduce expression of the indicated genes involved in metabolism and hepatic differentiation (Fig. 3a). Moreover, GSK-J4 strongly reduced both Cyp3A4 and Albumin at the protein levels in dHepaRG cells (Fig. 3b). We then evaluated whether demethylation activity of JMJD3 and UTX directly affect transcriptional regulation hepatic specific genes by modulating their promoter methylation status in dHepaRG. We performed a ChIP assay to study levels of H3K27me3 H3K27me3 together with the acetylation of lysines H4 (acH4) that is an epigenetic marker of transcriptional activation. We showed that binding of acetylated-Histone4 to both Albumin, Cyp3A4, HNF4 and CEBPb promoters, in response to GSK-J4 treatment, decreased (Fig. 3c left panels, and Fig. S3a left panels) and in parallel binding of H3K27me3 histone3 increased (Fig. 3c, right panels, and Fig. S3a right panels), indicating transcriptional repression. In addition, we demonstrated that GSK-J4 treatment modulate also common PRC2 target genes such as HOXA1 and CDKN2A (Fig. S3b).

To further demonstrate a role of JMJD3 and UTX methylases in hepatic differentiation, we assessed by FACS analysis the expression levels of CD49a-integrin, which is highly expressed in differentiated hepatocytes^31^, showing a reduction of its expression in GSK-J4 treated dHepaRG cells (Fig. 3d). Moreover, GSK-J4 dHepaRG treated cells lowered the expression of another marker of hepatic differentiation, CK19^32^, as shown in green by immunofluorescence assay (Fig. 3e). To support the results observed in dHepaRG cells we took advantage of human primary hepatocytes (PHH). In order to evaluate if GSK-J4 is able to revert the differentiated phenotype of PHH, we measured levels of secreted Albumin, by ELISA assay and Cyp3A4 activity, by a luminescent method. We showed that both secreted Albumin (Fig. 3f) and CyP3A4 activity (Fig. 3g) were reduced in PHH cells 48 and 96 h after GSK-J4 treatment as compared to control cells. These results demonstrated that GSK-J4 inhibition of JMJD3 and UTX H3K27 demethylase activity led to reduction of several hepatic differentiation markers in dHepaRG and PHH cells.

### GSK-J4 inhibition of JMJD3 and UTX H3K27 demethylase activity induced proliferation of dHepaRG cells

**Fig. 4:**
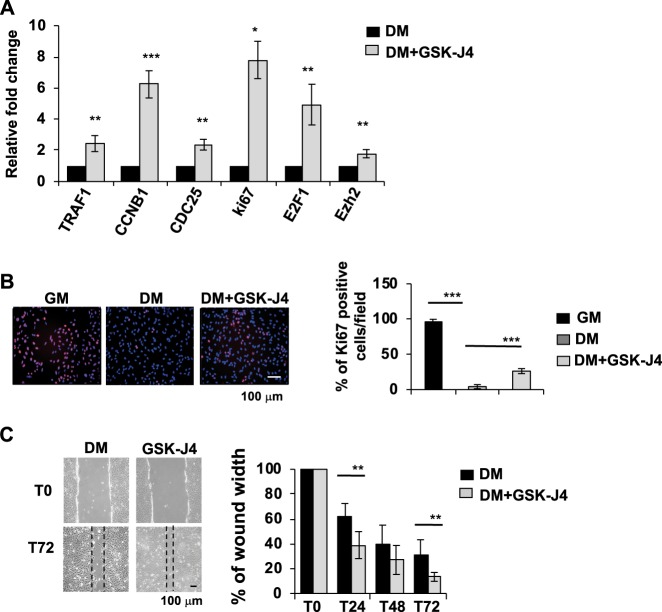
GSK-J4 treatment increased cell proliferation in dHepaRG cells. **a** Total RNA was extracted from dHepaRG cells treated with GSK-J4 for 24 h and qPCR analysis was performed using specific primers (Table S3). Amplification of GAPDH transcripts was used to normalize equal loading of each RNA samples. Histograms show the fold induction of treated cells (GSK-J4) versus untreated (DM). **b** Ki67 and Hoechst immunofluorescence of dHepaRG cells left untreated or treated for 48 h with GSK-J4 and compared to pHepaRG (GM) (left panels). Histograms indicate ki67 positive cells (red) over total number of cells (blue) expressed as percentage of the GM experimental point (right panel). **c** Scratch wound migration assay of dHepaRG treated with GSK-J4 for 24 h. After treatment the dimension of scratch area was measured, and measurement was repeated at 24, 48, 72 h after treatment. Representative images are showed in the left panels and histograms show percentage of wound width over the T0 experimental point. All results are expressed as fold induction from three independent experiments, bars indicate S.D.; Asterisks indicate *P*-value: *0.01 ≤ *P* < 0.05; **0.001 ≤ *P* < 0.01; ***P < 0.001

To confirm RNAseq. results we measured by qPCR the expression of selected genes involved in DNA replication and cell cycle pathways, as highlighted by KEGG analysis (Fig. 2c upper panel). We validated that inhibition of JMJD3 and UTX by GSK-J4 treatment was able to induce expression of TRAF1, CCNB1, CDC25A, MKI67, E2F1 and EZH2 genes in dHepaRG cells (Fig. 4a). To analyse protein levels of Ki67, a marker of cell proliferation, we performed an Immunofluorescence experiment. We showed that Ki67 protein expression was high in pHepaRG cells (GM) and was nearly undetectable in dHepaRG cells (DM), as expected. Interestingly, Ki67 increased after GSK-J4 treatment in dHepaRG cells as compare to DM cells (Fig. 4b).

To further study GSK-J4 effect on proliferation of dHepaRG cells we performed a scratch wound assay. Images of wound healing were taken immediately after scratching (T0 Fig. 4c) and after 24-48-72 h of GSK-J4 treatment (T24, T48, T72 Fig. 4c). We observed that differentiated cells after GSK-J4 treatment showed a higher proliferation rate already after 24 h of treatment, as demonstrated by more narrow wound width of GSK-J4 treated cells as compared to control cells (Fig. 4c).

These results demonstrated that GSK-J4 inhibition of JMJD3 and UTX is able to boost proliferation of dHepaRG cells.

### Release from GSK-J4 treatment rescue expression levels of proliferation marker genes

**Fig. 5:**
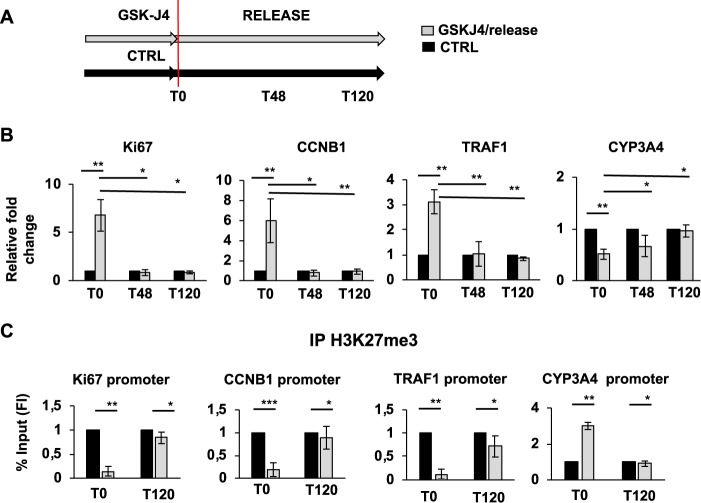
Release from GSK-J4 treatment rescues basal condition of dHepaRG cells. **a** Representative cartoon of the release experiment. DHepaRG cells were treated or not (CTRL) for 48 h with GSK-J4 and then shifted to differentiation medium (RELEASE) and harvested at the indicate times (T0, T48, T120 hours). **b** Total RNA from pHepaRG cells treated as in A were analyzed by qPCR. Histograms show fold induction of GSK-J4/release versus control cells (CTRL). **c** Anti-H3K27me3 immunoprecipitated chromatin from dHepaRG cells treated as in (**a**) were analyzed by qPCR using KI67, CCNB1, TRAF1 and CYP3A4 promoter selective primers (Table S3). % of input was calculated by Delta Ct analysis and expressed as fold induction of GSK-J4/release versus control cells (CTRL). All results are expressed as fold induction from three independent experiments, bars indicate S.D.; Asterisks indicate *P*-value: *0.01 ≤ *P* < 0.05; **0.001 ≤ *P* < 0.01; ***P < 0.001

To analyze if the proliferating activity of GSK-J4 has a long-term and cell transforming effect on dHepaRG phenotype, we performed a “release experiment”. After GSK-J4 treatment, cells were shifted to differentiation medium without GSK-J4 and harvested after 48 or 120 h (Fig. 5a). As shown, already 48 h after release from GSK-J4 treatment the expression level of cell proliferation marker genes MKI67, CCNB1 and TRAF1 returned to basal dHepaRG level. Moreover, also the expression of Cyp3A4 gene, that was reduced after GSK-J4, was restored to basal dHepaRG level after 120 h (Fig. 5b).

Accordingly with these results, we have performed a ChIP experiments to test H3K27me3 levels on cell cycle promoters. As we showed in Fig. 5c, 120 h after release from GSK-J4 treatment the H3K27me3 level on MKI67, CCNB1 and TRAF1 promoters returned to basal dHepaRG level. As expected, H3K27me3 level on Cyp3A4 promoter reduced after GSK_J4 treatment release (Fig. 5c). These results suggested that after the removal of GSK-J4 treatment dHepaRG cells readily arrest their proliferation and are able to re-induce a differentiated phenotype, demonstrating the reversible effect of the GSK-J4 treatment.

### GSK-126 is an anti-proliferative drug and induced differentiation of proliferating HepaRG cells

**Fig. 6:**
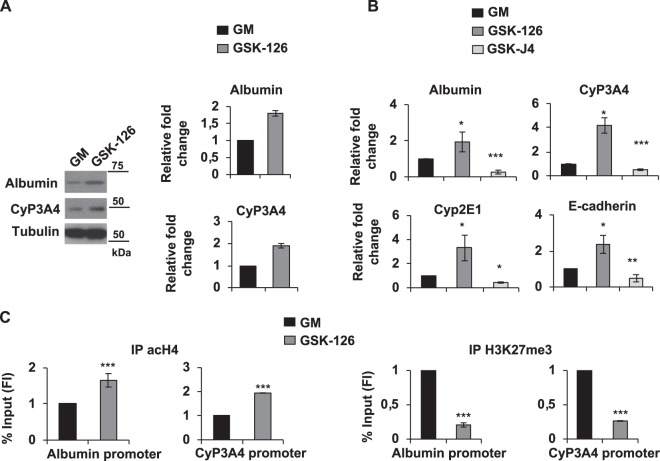
GSK-126 induced hepatic differentiation in pHepaRG cells. **a** Total protein lysates from pHepaRG cells (GM) treated or not for 48 h with GSK-126 were analyzed by immunoblot with the indicated antibodies (Table S4, left panels), histograms show densitometric analysis (right panels). **b** Total RNA from pHepaRG cells treated or not with GSK-J4 and GSK-126 for 48 h were analyzed by qPCR with indicated antibodies (Table S3). **c** Anti-acH4 and anti-H3K27me3 immunoprecipitated chromatin from pHepaRG cells treated as in (**a**) were analyzed by qPCR. All histograms show fold induction of treated (GSK-J4/GSK-126) versus control cells (GM). All results are expressed as fold induction from three independent experiments, bars indicate S.D.; Asterisks indicate *P*-value: *0.01 ≤ *P* < 0.05; **0.001 ≤ *P* < 0.01; ***P < 0.001

As shown in Fig. 1a, b, EZH2, JMJD3 and UTX were high at both the protein and transcript levels in pHepaRG cells. However, EZH2 strongly decreased during differentiation, becoming nearly undetectable in differentiated cells. To characterize EZH2 role in proliferating cells and to further study methylases and demethylases activity in the regulation of liver differentiation, we took advantage of GSK-126, a highly selective inhibitor of EZH2 H3K27-methyltransferase activity (Supplementary Fig. S1a, c). Inhibition of EZH2 methyltransferase activity by GSK-126 increased the expression of both Albumin and Cyp3A4 proteins in pHepaRG cells (Fig. 6a). Same results were observed by qPCR that showed an increase of Albumin, CyP3A4, CyP2E1, E-cadherin liver specific transcripts after GSK-126 treatment and a reduction of these transcripts after GSK-J4 treatment as compared to control cells (Fig. 6b). We confirmed these results in the hepatocellular carcinoma cell line HepG2 (Supplementary Fig. S2 and S4 panel a, b).

In order to study whether EZH2 directly affects the expression via methylation of Histone3, we next examined the chromatin changes of liver gene promoters in pHepaRG cells upon GSK-126 treatment. To this aim we performed a ChIP assay with H3K27me3 and acH4 specific antibodies. We demonstrated that after GSK-126 treatment Albumin and CyP3A4 promoters were enriched in acH4 proteins (Fig. 6c, left panels) and the binding of H3K27me3 to both promoters decreased (Fig. 6c, right panels), confirming epigenetically transcriptional activation of these genes after EZH2 inhibition. These results demonstrated that inhibition of EZH2 methyltransferase activity by GSK-126 is able to directly induce liver specific gene expression suggesting a role for EZH2 in the maintenance of a proliferative status in HepaRG cells.

### GSK-126 treatment inhibited proliferation of HepaRG cells

**Fig. 7:**
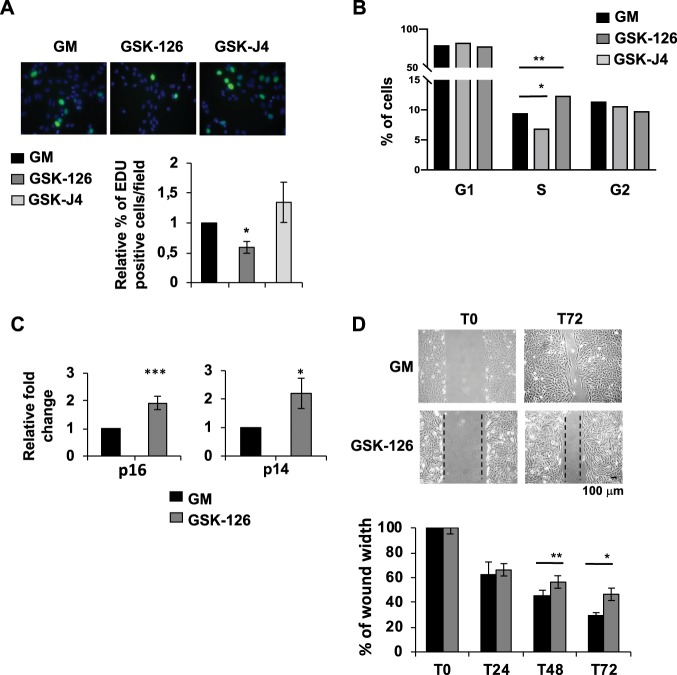
GSK-126 inhibited cell proliferation in pHepaRG cells. **a** pHepaRG were treated with GSK-126 and GSK-J4 for 72 h. 2 h after incubation with EdU, the cells are fixed and stained with Click-iT kit. Dividing cells incorporated with EdU are shown in green, total cells counterstained with Hoechst are in blue (upper images). Number of EdU positive cells were calculated over total cells and expressed as fold induction vs pHepaRG (GM) cells. **b** FACS cell cycle analysis after PI staining of pHepaRG treated as in (**a**). Histograms show % of cells in different phases of cell cycle (**c**) Total RNA from pHepaRG treated with GSK-126 for 48 h were analyzed by qPCR with indicated primers (Table S3). Histograms show fold induction of treated (GSK-126) versus untreated cells (GM). **d** Scratch wound migration assay of pHepaRG treated with GSK-126 for the indicated time (T0, T24, T48, T72). Results are expressed as % of wound width over the T0 experimental point. Representative images are showed in the upper panels. **a**–**d** Histograms are expressed as fold induction from three independent experiments, bars indicate S.D.; Asterisks indicate *P*-value: *0.01 ≤ *P* < 0.05; **0.001 ≤ *P* < 0.01; ***P < 0.001

Considering the pro-differentiative effect of GSK-126 on pHepaRG cells and the anti-differentiative outcome of GSK-J4, we sought to better study their role in modulating proliferation of these cells. We performed an EdU assay to detect and quantify cell proliferation using fluorescence microscopy. We showed that inhibition of EZH2 by GSK-126 reduced HepaRG cell ability to divide, while GSK-J4 didn’t have any significative effect (Fig. 7a). Indeed, we observed by FACS analysis after PI incorporation that GSK-126 inhibited S phase of pHepaRG cells and GSK-J4 slightly but significantly enhanced it (Fig. 7b). We analysed transcript levels of p16 and p14, two alternatively spliced variants encoded by CDKN2A (Cyclin-Dependent Kinase 2 Inhibitor A), that plays an important role in cell cycle regulation by inhibiting the progression from G1 to S phase. Accordingly, we showed that both transcripts are upregulated upon GSK-126 exposure (Fig. 7c). Similar results were obtained in HepG2 cells by FACS analysis after PI staining, qPCR evaluation of p16 and p14 expression and EDU assay (Supplementary Fig. S4, panels c, d, e).

To further confirm EZH2 role in hepatic cell proliferation we analyzed pHepaRG cells growth rate by a scratch wound healing assay. Cells were treated immediately after scratching (T0) and images were captured at 24, 48, 72 h after treatment (T24, T48, T72). We observed that inhibition of EZH2 with GSK-126 decreased the migration and growth rate of pHepaRG cells already at 48 h after treatment (Fig. 7d). These data demonstrated that GSK-126 display an anti-proliferative effect.

## Discussion

Although epigenetic mechanisms play important roles in differentiation and development of human embryonic stem cells^32,33^, the epigenetic factors that are primarily responsible for establishing a differentiated state are currently unknown. In this study, we revealed that GSK-J4 inhibitory activity on histone demethylase JMJD3 and UTX led to retro-differentiation of dHepaRG cells through the activation of proliferating genes and the inhibition of genes specific for liver differentiation.

Firstly, we have observed in dHepaRG cells that expression level of these methylase/demethylase enzymes are differently modulated during differentiation: in dHepaRG cells both EZH2 protein and transcripts are strongly reduced, whereas JMJD3 and UTX demethylases expression levels are not affected. According to these results, H3K27me3 was significantly reduced in dHepaRG cells and GSK-J4 treatment restored H3K27me3 to pHepaRG cells level. By optical microscope imaging, we observed that dHepaRG cells changes their phenotype 48 h after GSK-J4 treatment. These impressive results led us to perform a genome wide analysis to better understand the GSK-J4 treatment effect on dHepaRG retro-differentiation. Interestingly, we could show by RNA sequencing that transcriptional expression signature of pHepaRG versus dHepaRG and GSK-J4 treated dHepaRG cells paralleled the observed morphology phenotype. Indeed, pHepaRG, dHepaRG and dHepaRG + GSK-J4 samples clusterized differently, and GSK-J4 treatment shifted dHepaRG cells RNA expression signature to proliferating cells profile, as shown by PCA analysis and Heat map Hierarchical clustering.

Kegg-GO analysis of GSK-J4 profiles versus dHepaRG cells revealed downregulated pathways linked to metabolism and among these there are also genes involved in hepatic differentiation such as cytochrome P450 proteins (CYP), aldehyde dehydrogenase family of proteins (ADH) and albumin, suggesting a role of JMJD3 and UTX in the maintenance of hepatic cell differentiation state. We performed a FACS analysis using a specific anti-CD49a that recognizes an integrin expressed in human hepatocyte and an immunofluorescence assay by anti-CK19 antibody that recognizes a cytokeratin 19 preferentially expressed in biliary cells. Thus, we showed that JMJD3 and UTX have an important role in maintenance of both hepatocyte and biliary cell differentiation.

Moreover, we observed that GSK-J4 was able to stimulate cell proliferation, survival and inflammation pathways. It has been demonstrated that IL6 (interleukin 6) and TNFα (tumor necrosis factor alpha) receptor 1 (Tnfrsf) are essential for liver regeneration and that NFκB and AP-1 transcriptional activity is critical for initiation of liver regeneration^34^. Thus, the activation of an inflammatory pathway by GSK-J4 might be responsible for induction of cell proliferation in term of early liver regeneration response. Moreover, it has also been shown that activation of TNFα, IL6, and TGFß signaling pathways directs the retro-differentiation of dHepaRG into bipotent progenitors^35^. Indeed, in our cell model we have observed that after IL6 treatment Albumin and CYp3A4 transcripts significantly decreased (Supplementary Fig. S4), as we observed after GSK-J4 treatment. Thus, our results could suggest that GSK-J4 treatment is able to epigenetically activate inflammatory pathways together with cell proliferation and these transcriptional changes could lead to an early liver regeneration.

Although we observed that GSK-J4 treatment activated pathways involved in transcriptional mis-regulation in cancer, we demonstrated that several epithelial mesenchymal transition (EMT) genes, that are activated in many cancers^36^, such as SNAIL, TWIST and ZEB1 and several genes involved in beta-catenin pathways, chromatin remodeling and angiogenesis, that are specifically upregulated during HCC tumorigenesis^37,38^, didn’t change their expression level after GSK-J4 treatment, as shown in Supplementary Table S2. Thus, we could hypothesize that the GSK-J4 induced proliferation leads to liver regeneration and survival, rather than oncogenic transformation.

Conversely, inhibition of EZH2 activity by GSK-126 treatment of proliferating HepaRG cells was able to arrest liver proliferation and increased Albumin and CYP3A4 expression level, according to previous papers^39^. Hence, several EZH2 inhibitors have been developed and are currently on pre-clinical studies and clinical trials for cancer therapy including hepatocellular carcinoma^40^.

Finally, by GSK-J4 “release experiment” we demonstrated that GSK-J4 is not able to induce a persistent cell proliferation, but already after 48 h of GSK-J4 release cells stopped to proliferate and return to a differentiated phenotype. It could be really interesting to further investigate a possible therapeutic application of GSK-J4 in liver regeneration since our results suggest that GSK-J4 epi-drug induce a reversible proliferation during treatment without cancer transformation and long term/irreversible effect on differentiation status.

Altogether these results demonstrated an important role of JMJD3/UTX/EZH2 in regulation of hepatic proliferation and differentiation, showing that modulation of their activities by epi-drugs is able to control hepatic cell plasticity.

## Supplementary information

Supplementary Material
