# Supplementary material for: EZH2, JMJD3, and UTX epigenetically regulate hepatic plasticity inducing retro-differentiation and proliferation of liver cells
Source: Cell Death Dis. 2019 Jul 8;10(7):518. doi: 10.1038/s41419-019-1755-2 (PMC6614397; doi:10.1038/s41419-019-1755-2)
Supplement: Supplementary file 1 — Supplementary Material [file 41419_2019_1755_MOESM1_ESM.pdf]

EZH2, JMJD3 and UTX epigenetically regulate hepatic plasticity inducing retro-differentiation and proliferation of liver cells.

Natalia Pediconi[1], Debora Salerno[1], Leonardo Lupacchini[2], Annapaola Angrisani[3] Giovanna Peruzzi[1], Enrico De Smaele[4], Massimo Levrero[1,5,6,7] and Laura Belloni[1].

1 Center for Life Nano Science@Sapienza, Istituto Italiano di Tecnologia, Rome, Italy
2 IRCCS San Raffaele Pisana, Rome, Italy
3 Dept. Molecular Medicine, Sapienza University of Rome, Italy
4 Dept. of Experimental Medicine, Sapienza University of Rome, Italy
5 Cancer Research Center of Lyon (CRCL), UMR INSERM U1052 - CNRS 5286, Lyon, France
6 Dept. of Internal Medicine and Medical Specialties, Sapienza University of Rome, Italy
7 Hepato-Gastroenterologie, Hopital de la Croix-Rousse, Hospices Civils de Lyon, Lyon, 69004, France

Corresponding author: Laura Belloni, viale Regina Elena 291, 00161 Rome, Italy, tel+39 0649255664, laura.belloni@gmail.com

Table of contents

Supplementary Methods

Supplementary Figures

Tables

Supplementary Methods

FACS analysis. To quantify cell viability after drugs treatment cells were stained with Fixable Viability Dye eFluor 780 (affymetrix eBioscience 65-0865). To evaluate cell cycle progression propidium iodide (PI) fluorescent intercalating agent was used according to the manufacture's instructions. To measure hepatic differentiation CD49a antibody (Biolegend cat.n. 328310) staining was performed. For each experiment, Fluorescence-activated cell sorting (FACS) analysis was performed using BD LSRFortessa and with DIVA software (BD Biosciences, San Jose, CA, USA). The cells were first visualized on dimension parameters using forward vs side scatter (FSC vs SSC) and upon different laser excitation fluorescence of each specific channel was detected. In each experiment, a total of 20000 cells per sample were acquired. Data were analyzed using FlowJo software (FlowJo LLC data analysis software, Ashland, OR, USA).

RNA-sequencing, library preparation, sequencing and bioinformatics analysis.

Total RNA from pHepaRG, dHepaRG and dHepaRG treated with GSK-J4 25 micromolar were extracted with Mirneasy Mini Kit from Qiagen (cat.no. 217004).

TruSeq Stranded Total RNA (Human) HT Sample Prep kit (Illumina, San Diego, CA) has been used for library preparation following the manufacturer's instructions, starting with good quality RNA (R.I.N. >7) as input. Purification steps have been performed by using 1X Agencourt AMPure XP beads. Both RNA samples and final libraries were quantified by the Qubit 2.0 Fluorometer (Invitrogen, Carlsbad, CA) and quality tested by Agilent 2100 Bioanalyzer RNA Nano assay (Agilent technologies, Santa Clara, CA). Libraries were then processed with Illumina cBot for cluster generation on the flowcell, following the manufacturer's instructions and sequenced on paired-end 125 bp mode on HiSeq2500 (Illumina, San Diego, CA). The CASAVA 1.8.2 version of the Illumina pipeline was used to processed raw data for both format conversion and de-multiplexing.

Three biological replicates were analyzed for each condition, resulting in 9 samples. Sequencing generated on average 53.5 M of reads/sample (min 43.2 M, max 60.2 M). After trimming with ERNE1 and removal of adapter sequences with Cutadapt2 reads were aligned on UCSC hg19 reference with STAR3, using default parameters. Median alignment rate was 88%. Gene expression, i.e. the relative abundances of transcripts was estimated by StringTie4 using default parameters. Pair-wise differential expression analysis was performed by DESeq25.

Log2 Fold change (activated genes >+0.58; repressed genes <-0.51) and p-value (≤ 0.05) for pair-wise sample comparison were calculated to evaluate differentially expressed genes which were carried out by further KEGG signaling pathway analysis (Kyoto Encyclopedia of Genes and Genomes) using DAVID on line tools (Database for Annotation, Visualization and Integrated Discovery, https://david.ncifcrf.gov/). A P value ≤ 0.05 was considered statistically significant. Unsupervised hierarchical clustering was performed by average linkage algorithm on selected differentially expressed gene lists using MultiExperiment Viewer (MeV, http://mev.tm4.org/).

Supplementary Figures

Figure S1

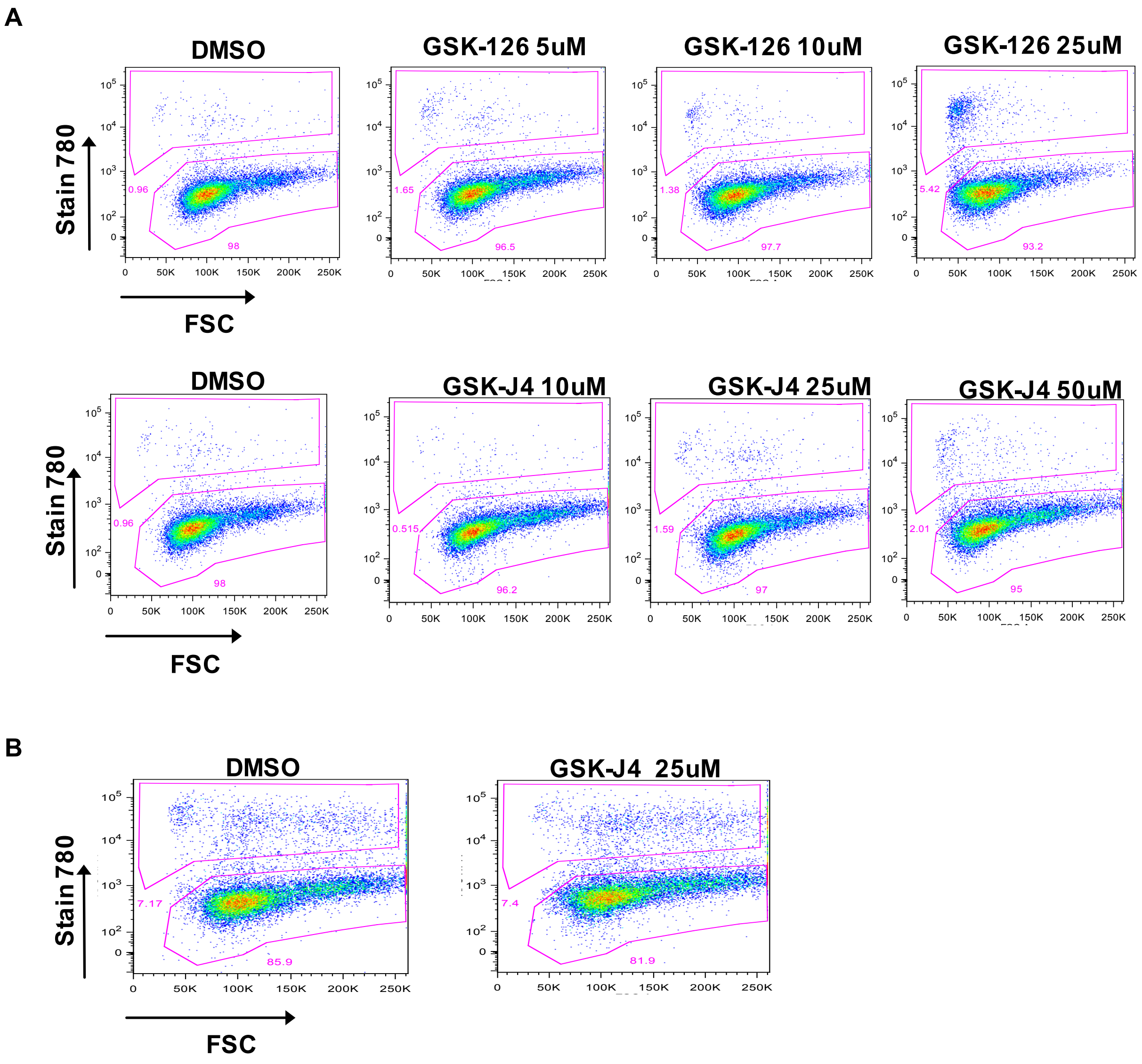

Figure S1. HepaRG cells viability after GSK-J4 and GSK-126 treatment. A) pHepaRG cells were treated or not with GSK-J4 and GSK-126 for 72 hours at the indicated concentrations. Viability was tested by FACS analysis after staining with Fixable Viability Dye eFluor™ 780. Cells are shown based on the positivity for the dye (Stain 780) versus FSC parameter. **B)** dHepaRG cells were treated with GSK-J4 for 48 hours at the indicated concentration. Viability was tested by FACS analysis as in A.

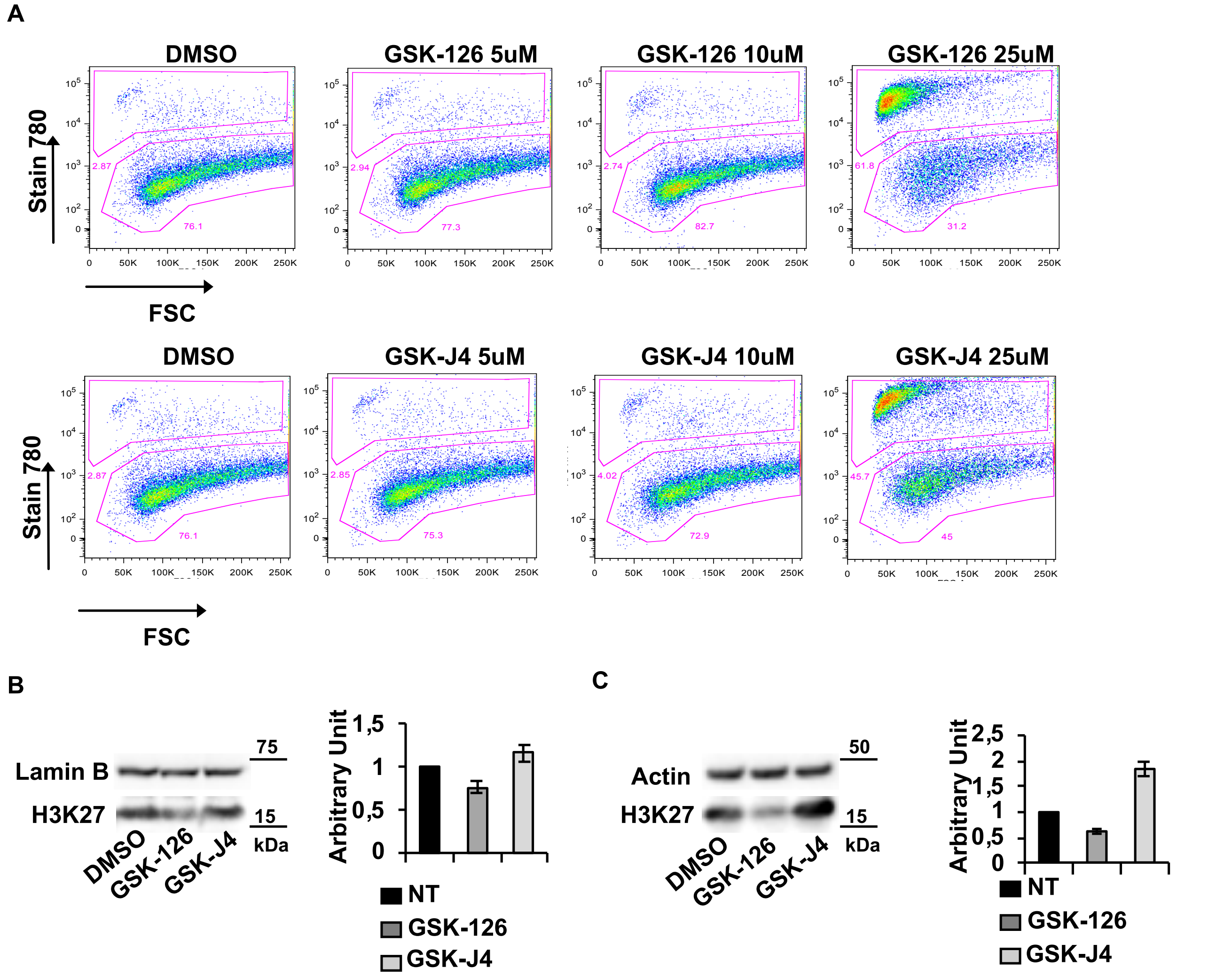

Figure S2. HepG2 cells viability after GSK-J4 and GSK-126 treatment and their effect on H3K27 residues in liver cell lines. A) HepG2 cells were treated or not with GSK-J4 or GSK-126 for 48 hours at the indicated concentrations. Viability was tested by FACS analysis after staining with Fixable Viability Dye eFluor™ 780. Cells are shown based on the positivity for the dye (Stain 780) versus FSC parameter. **B)** Nuclear protein lysates were extracted from pHepaRG cells treated or not with GSK-J4 or GSK-126 for 72 hours and analyzed by immunoblot with the indicated antibodies (Table S4). Histograms show densitometric analysis. **C)** Nuclear protein lysates were extracted from HepG2 cells treated as in A and analyzed by immunoblot with the indicated antibodies (Table S4). Histograms show densitometric analysis.

Figure S3

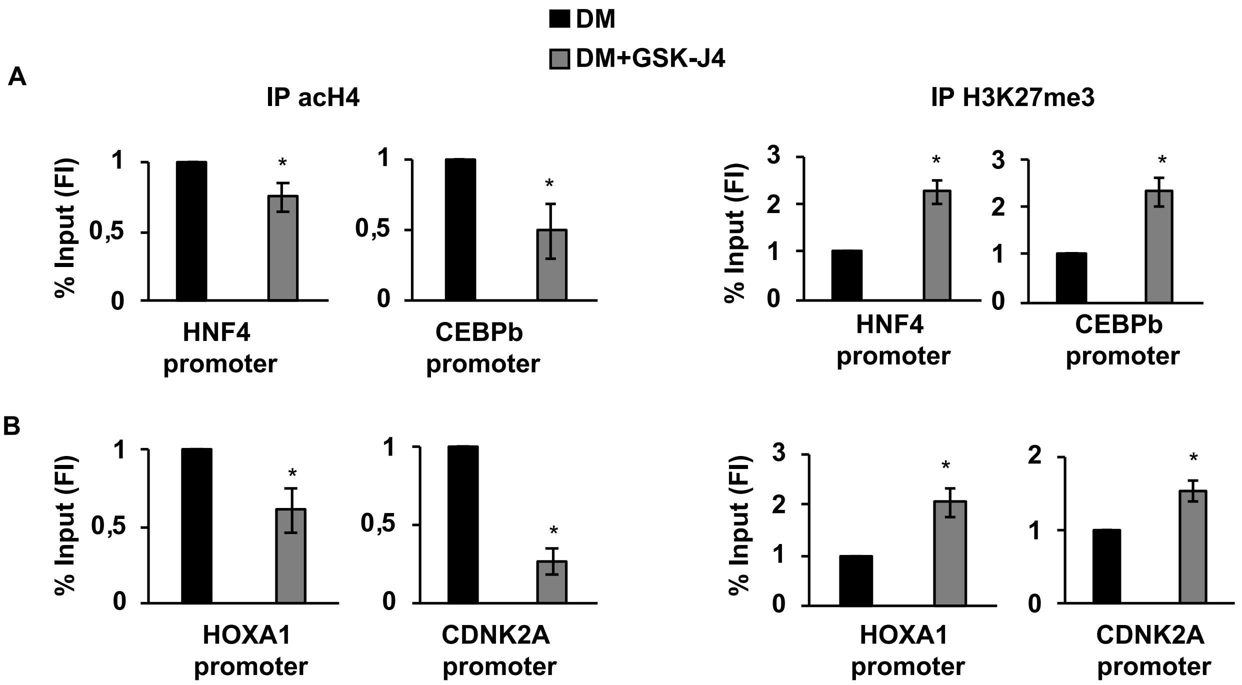

Figure 3. ChiP assay in dHepaRG cells after GSK-J4 treatment. **A, B)** Cross-linked chromatin was extracted from dHepaRG cells treated or not for 48 hours with GSK-J4 and immunoprecipitated with a relevant control IgG or specific anti-AcH4 and anti-K27me3 antibodies (respectively left and right panels). Immunoprecipitated chromatin samples were analyzed by qPCR using HNF4 and CEBPb promoter as showed in panel A and HOXA1 and CDKN2A promoter in panel B. Histograms show % of input calculated by Delta Ct analysis and expressed as fold induction (mean) of treated cells (GSK-J4) versus untreated (DM) from three independent experiments; bars indicate S.D.; asterisks indicate p-value: * 0.01 ≤ P < 0.05; ** 0.001 ≤ P < 0.01; *** P < 0.001.

Figure S4

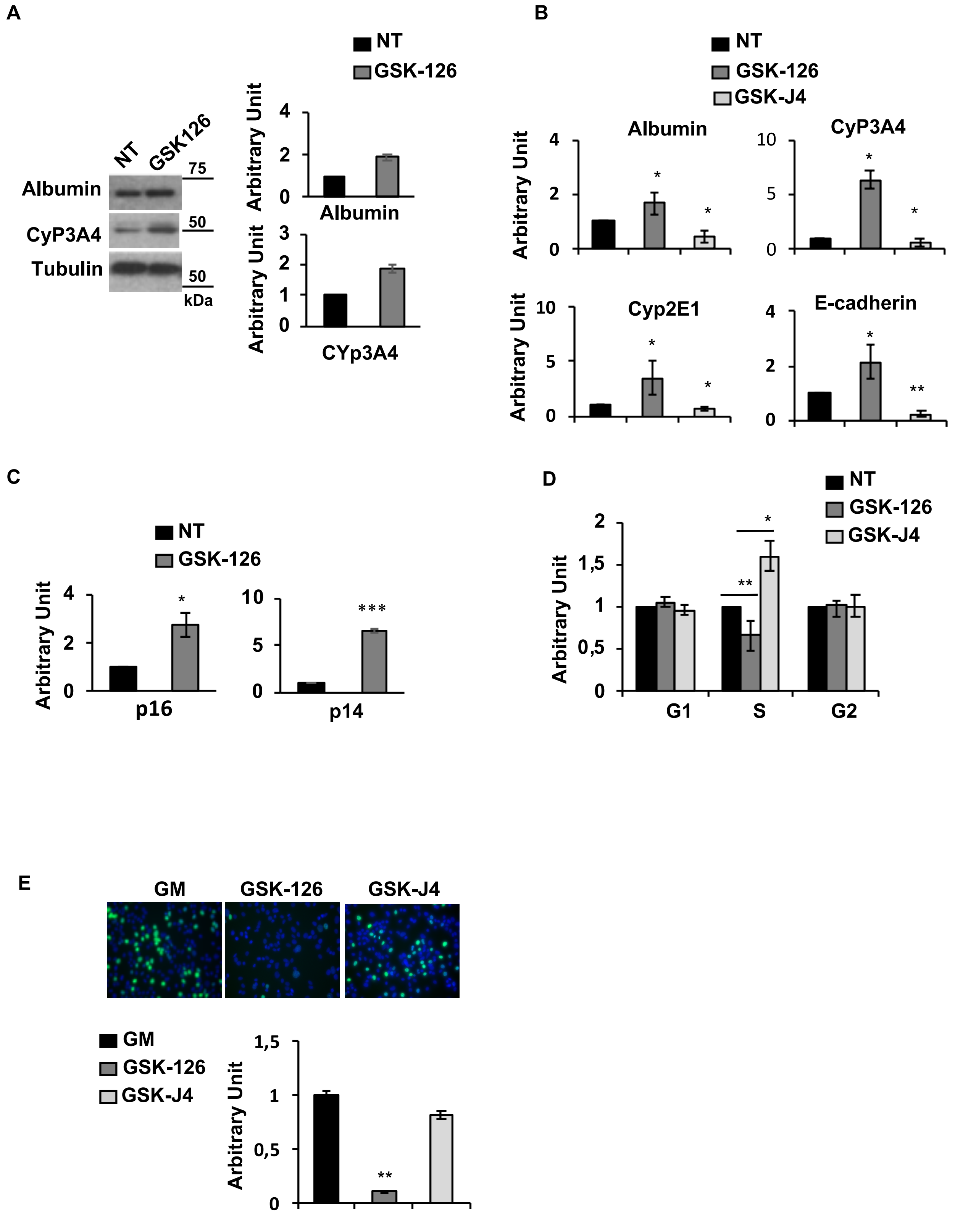
A
NT
GSK-126
NT
GSK126
Albumin
CyP3A4
Tubulin
75
50
50
kDa
Arbitrary Unit
4
2
0
Albumin
3
2
1
0
CYp3A4
B
NT
GSK-126
GSK-J4
Albumin
CyP3A4
Cyp2E1
E-cadherin
Arbitrary Unit
10
5
0
*
**
C
NT
GSK-126
Arbitrary Unit
p16
p14
***
D
NT
GSK-126
GSK-J4
Arbitrary Unit
2
1,5
1
0,5
0
G1
S
G2
E
GM
GSK-126
GSK-J4
1,5
1
0,5
0
**

Figure S4. Inhibition of Ezh2/JMJD3/UTX activity in HepG2 cell line. A) Total protein lysates were extracted from HepG2 cells treated for 48 hours with GSK-126 and analyzed by immunoblot analysis with the indicated antibodies (Table S4), left panels. Histograms represent densitometric analysis, right panels. **B and C)** Total RNA were extracted from HepG2 cells treated GSK-126 or GSK-J4 for 48 hours, qPCR analysis was performed using the indicated primers (Table S3). Amplification of GAPDH transcripts was used to normalize equal loading of each RNA samples. **D)** FACS analysis after PI staining of HepG2 cells treated as in B. Histograms represent % of cells in G1, S and G2 phases expressed as fold induction of treated versus untreated (NT) cells. **E)** EDU assay of HepG2 cells treated as in B. After incubation with EdU, the cells are fixed and stained with Click-iT kit. Dividing cells incorporated with EdU are shown in green, total cells counterstained with Hoechst are in blue (upper images). Number of EdU positive cells were calculated over total cells and expressed as fold induction vs pHepaRG (GM) cells. Results are mean from three independent experiments; bars indicate S.D.; asterisks indicate p-value: * 0.01 ≤ P < 0.05; ** 0.001 ≤ P < 0.01; *** P < 0.001.

Figure S5

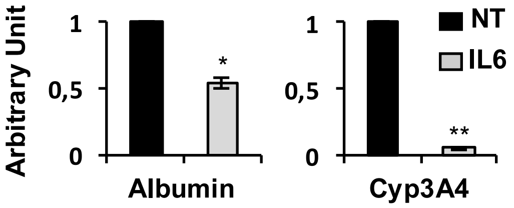

Figure S5. IL6 treatment of dHepaRG decreased hepatic liver markers. Total RNA were extracted from dHepaRG cells treated with IL6 for 48 hours, qPCR analysis was performed using the indicated primers (Table S3). Amplification of GAPDH transcripts was used to normalize equal loading of each RNA samples. Histograms show fold induction of treated (IL6) versus untreated cells (NT). Results are mean from three independent experiments; bars indicate S.D.; asterisks indicate p-value: * 0.01 ≤ P < 0.05; ** 0.001 ≤ P < 0.01; *** P < 0.001.

TABLE S1

TNF signaling pathway		GSK-J4 versus DMSO
	log2FoldChange	pvalue
IL6	2,954256785	6,72E-41
TRAF1	1,571765942	4,22E-20
CSF1	1,025821843	6,66E-20
CCL2	1,456071707	2,68E-17
IL1B	1,662121726	1,13E-13
CXCL1	0,925508094	1,10E-12
CXCL2	1,080963076	1,41E-12
MMP3	2,023846605	1,00E-11
CCL20	1,775279777	9,54E-10
LIF	1,764823365	1,59E-08
MAP3K8	0,635175121	2,46E-07
JUN	0,664466305	1,51E-06
NFKB1	0,51227046	2,57E-06
PTGS2	1,158617713	2,30E-05
CXCL3	0,729878898	4,59E-05
TNFRSF1B	0,623480869	0,004242132

NF-kappa B signaling pathway GSK-J4 versus DMSO		
	log2FoldChange	pvalue
MMP14	1,591329968	2,38E-80
IRAK1	1,057374133	3,04E-33
CXCL8	1,525082568	1,45E-30
IL18R1	1,510900745	3,48E-27
TNFAIP3	1,050950353	2,41E-24
ICAM1	1,218899482	4,36E-21
SOCS3	1,20514565	7,39E-20
LBP	1,623614699	1,21E-13
BIRC3	0,602429745	4,97E-09
BIRC3	0,602429745	4,97E-09
RELB	0,787140636	1,46E-06
LYN	0,553674865	2,20E-06
BCL2A1	1,354216485	5,83E-06
RPS6KA4	0,624601646	2,64E-05
MAP2K3	0,618288871	5,60E-05
CREB5	0,773916092	0,00126468
TNFRSF11A	0,773302616	0,00223827

Table S1. Inflammatory genes are upregulated after GSK-J4 treatment in dHepaRG cells. Expression levels of the indicated genes in GSK-J4 treated versus control dHepaRG cells were analyzed by RNA sequencing. Mean of three indipendent experiments are expressed as log2 fold change.

TABLE S2

Chromatin remodeling		
	**log2FoldChange**	**pvalue**
**ARID1B**	-0,232442421	0,03806123
**ARID1A**	0,034259336	0,82327292

BETA-CATENIN pathway		
	**log2FoldChange**	**pvalue**
**LGR5**	0,232879825	0,44294069
**EGFR**	0,196270132	0,07458786
**MYC**	0,001918379	0,98437632
**PROM1**	0,1797932	0,56601217
**CTNNB1**	0,297286995	8,28E-05
**AXIN2**	0,580230199	1,32E-05
**ADAM10**	0,006191783	0,94340982
**LEF1**	0,595767463	0,02848575
**FZD7**	0,353556704	0,23062515
**DKK1**	0,145963052	0,2693014

Epithelial-Mesenchymal Transition pathways		
	**log2FoldChange**	**pvalue**
**SNAI1**	0,032027506	0,89074591
**TWIST1**	-0,372051817	0,02129283
**ZEB1**	0,001734684	0,98760677

Angiogenic protein		
	**log2FoldChange**	**pvalue**
**VEGFA**	-0,497836446	0,00026402
**VEGFB**	-0,466284444	0,1083891
**VEGFC**	0,358513109	0,2615705
**PDGFRB**	-0,179443743	0,52688666
**MET**	0,226353338	0,03031959
**HGF**	0,415017308	0,19008406
**ANGPT1**	-0,729326093	4,11E-14
**ANG**	-0,589108545	0,02307619

Table S2. HCC marker genes are not modulated after GSK-J4 treatment in dHepaRG cells. Expression levels of the indicated genes in GSK-J4 treated versus control dHepaRG cells were analyzed by RNA sequencing. Mean of three indipendent experiments are expressed as log2 fold change.

TABLE S3

NAME	OLIGO SEQUENCE
ADH1B FOR	CCCGGAGAGCAACTACTGC
ADH1B REV	AACCAGTCGAGAATCCACAGC
ALBUMIN FOR	TGCTTGAATGTGCTGATGACAGG
ALBUMIN REV	AAGGCAAGTCAGCAGGCATCTCATC
CCNB1 FOR	AGTTATGCAGCACCTGGCTA
CCNB1 REV	TTAGCATGCTTCGATGTGGC
CD49a FOR	ATTCAATGACTTTCAGCGGC
CD49a REV	GCCAACTAACGGAGAACCAA
CDC25A FOR	TACCTCAGAAGCTGTTGGGATGT
CDC25A REV	CGAGATACAGGTCTTACTGGCTT
CYP2E1 FOR	TTGAAGCCTCTCGTTGACCC
CYP2E1 REV	CGTGGTGGGATACAGCCAA
CYP3A4 FOR	CTTCATCCAATGGACTGCATAAAT
CYP3A4 REV	TCCCAAGTATAACACTCTACACAGACAA
E-CADHERIN FOR	GAACGCATTGCCACATACAC
E-CADHERIN REV	ATTCGGGCTTGTTGTCATTC
E2F1 FOR	CTGCAGAGCAGATGGTTATG
E2F1 REV	GCTCTTAAGGGAGATCTGAAAG
EZH2 FOR	TTAACGGTGATCACAGGATAGG
EZH2 REV	AGGTAGCAGATGTCAAGGGATTT
FMO3 FOR	AATGGTTATCCTTGGGACATGC
FMO3 REV	AGATGGCTGTCGGTAAATTGTTC
GAPDH FOR	TGACAACTTTGGTATCGTGGAAGG
GAPDH REV	AGGGATGATGTTCTGGAGAGCC
HNF4 FOR	TGCGACTCTCCAAAACCCTC
HNF4 REV	ATTGCCCATCGTCAACACCT
JMJD3 FOR	CCTCGAAATCCCATCACAGT
JMJD3 REV	GTGCCTGTCAGATCCCAGTT
KRT19 FOR	TGAGTGACATGCGAAGCCAAT
KRT19 REV	CTCCCGGTTCAATTCTTCAGTC
MKI67 FOR	GACTTTGGGTGCGACTTGAC
MKI67 REV	CAACTCTTCCACTGGGACGAT
P14 FOR	CCCTCGTGCTGATGCTACTG
P14 REV	ACCTGGTCTTCTAGGAAGCGG
P16 FOR	GAAGGTCCCTCAGACATCCCC
P16 REV	CCCTGTAGGACCTTCGGTGAC
SNAIL1 FOR	TCTCTAGGCCCTGGCTGCTA
SNAIL1 REV	GACATCTGAGTGGGTCTGGAG
TRAF1 FOR	TCCTGTGGAAGATCACCAATGT
TRAF1 REV	GCAGGCACAACTTGTAGCC
TWIST1 FOR	TTCTCGGTCTGGAGGATGGA
TWIST1 REV	AATGACATCTAGGTCTCCGGC
UTX FOR	AGCTGAAGGAAAAGTGGAGTCT
UTX REV	AGGCAGCATTCTTCCAGTAGT
ZEB1 FOR	ATGACCTGCCAACAGACCAG
ZEB1 REV	CCTTTCCTGTGTCATCCTCCC
ALBUMIN PROMOTER FOR	GGCAGCCAATGAAATACAAAGA
ALBUMIN PROMOTER REV	TAGGACAAACGGAGGGAAATTAG
CYP3A4 PROMOTER FOR	TGATGGCTCTCATCCCAGACT
CYP3A4 PROMOTER REV	AGAAGAGGAGCCTGGACAGTT

HNF4 PROMOTER FOR	TGCTCACATCTCGTTACCTGA
HNF4 PROMOTER REV	TTTCTCTCCTTTCCGGCACA
CEBPb PROMTER FOR	ATGTGCAGCCCACAACGTA
CEBPb PROMOTER REV	CAATCCATGAAGGGTGTCGC
HOXA1 PROMOTER FOR	TTTCTTGCCCTCGTCTCCTT
HOXA1 PROMOTER REV	GTGAGGAGCGCATAGAACCG
CDKN2A PROMOTER FOR	GCCATACTTTCCCTATGACACC
CDKN2A PROMOTER REV	CCGCGATACAACCTTCCTAAC

TABLE S4

Anti-JMJD3	Abcam cat.n.154126
Anti-UTX	Cell Signaling cat.n. 33510
Anti-Ezh2	Cell Signaling cat.n. 5246
Anti E2F1	Santa Cruz cat.n. 56662
Anti-Albumin	Cell Signaling cat.n. 4929
Anti Cyp3A4	Cell Signaling cat.n. 13384
Anti-ActinB	Santa Cruz cat.n. 1616
Anti-Ki67	NG Markers, RM-9106-S
Anti-CD49a	Biolegend cat.n. 328310
Anti-CK19	Dako M0772
Anti-H3K27me3	Abcam 6002
Anti-H3	Abcam 1791
Anti-Ach4	Millipore cat.n. 06866
Anti-IgG	Cell Signaling cat.n. 27478
Anti-Tubulin	Neo Markers MS-581-P1
